# High-Performance *Cladophora*-Algae-Based Paper for Honeycomb Core in Sandwich-Structured Composite: Preparation and Characterizations

**DOI:** 10.3390/polym15061359

**Published:** 2023-03-09

**Authors:** Yati Mardiyati, Anna Niska Fauza, Steven Steven, Onny Aulia Rachman, Tatacipta Dirgantara, Arief Hariyanto

**Affiliations:** 1 Materials Science and Engineering Research Group, Faculty of Mechanical and Aerospace Engineering, Institut Teknologi Bandung, Jl. Ganesha No. 10, Bandung 40132, Indonesia; 2 Lightweight Structure Research Group, Faculty of Mechanical and Aerospace Engineering, Institut Teknologi Bandung, Jl. Ganesha No. 10, Bandung 40132, Indonesia; 3 Energy Conversion Research Group, Faculty of Mechanical and Aerospace Engineering, Institut Teknologi Bandung, Jl. Ganesha No. 10, Bandung 40132, Indonesia

**Keywords:** cellulose fibers, biomaterials, biocomposites, biodegradability, *Cladophora* algae, honeycomb core, sandwich-structured composites

## Abstract

Cellulose is classified as one of the most abundant biopolymers in nature. Its excellent properties have gained a lot of interest as an alternative material for synthetic polymers. Nowadays, cellulose can be processed into numerous derivative products, such as microcrystalline cellulose (MCC) and nanocrystalline cellulose (NCC). MCC and NCC have demonstrated outstanding mechanical properties owing to their high degree of crystallinity. One of the promising applications of MCC and NCC is high-performance paper. It can be utilized as a substitute for the aramid paper that has been commercially used as a honeycomb core material for sandwich-structured composites. In this study, MCC and NCC were prepared by extracting cellulose from the *Cladophora* algae resource. MCC and NCC possessed different characteristics because of their distinct morphologies. Furthermore, MCC and NCC were formed into a paper at various grammages and then impregnated with epoxy resin. The effect of paper grammage and epoxy resin impregnation on the mechanical properties of both materials was studied. Then, MCC and NCC paper was prepared as a raw material for honeycomb core applications. The results showed that epoxy-impregnated MCC paper outperformed epoxy-impregnated NCC paper with a compression strength of 0.72 MPa. The interesting result from this study is that the compression strength of the MCC-based honeycomb core was comparable to the commercial ones despite being made of a natural resource, which is sustainable and renewable. Therefore, cellulose-based paper is promising to be used for honeycomb core applications in sandwich-structured composites.

## 1. Introduction

A sandwich-structured composite is a special class of composite materials which consists of face sheets or skins and a core embedded between them [1,2]. The application of sandwich-structured composites has significantly increased due to their suitability in developing lightweight structures with high mechanical performance and they have become the first choice in various applications, especially in the structural field [3,4]. Sandwich-structured composites have begun to be widely used in vast areas, ranging from satellites [5] and aircraft wings [6], as well as marine [7], transportation [8], sport [9], and drone or military applications [1]. Using sandwich-structured composites in transportation applications results in a lighter vehicle with better fuel efficiency and payload capacity [10,11].

The core material plays an important role in the sandwich-structured composite. When the sandwich-structured composite is subjected to a load, tensile and compressive stress will be distributed to the skin, and the core will resist the shear stress. This mechanism results in the sandwich-structured composite’s advantage: a high bending stiffness and strength-to-weight ratio [12,13]. The core material that has been widely used in sandwich-structured composites is the honeycomb core type [4]. The honeycomb core is a cellular material that imitates the bee hive’s structure in nature, which was shown to have the most efficient material use, while the hexagonal structure gives relatively high out-of-plane compression properties [14,15,16]. One of the most popular commercial honeycomb core types to use is phenolic-impregnated aramid paper honeycomb due to its excellent properties [17]. It provides high out-of-plane mechanical properties [13], flame resistance, strong resistance to chemical attack, and corrosion resistance [18]. However, despite its excellent properties, aramid paper is made of crude oil-based synthetic materials [19]. The production of synthetic materials has become a global environmental issue in the last decade that tends to be a disadvantage to the environment. This is due to its highly complex process that leads to high energy consumption and greenhouse gas emission [20]. Thus, the urgent need to develop renewable, sustainable, and environmentally friendly materials has become a concern in the past few years. Therefore, alternative materials are needed to replace aramid as the raw material for honeycomb core manufacturing. The alternative material that is promising to be utilized as a raw material for an environmentally friendly honeycomb core is cellulose.

Cellulose is the most abundant natural polymer, obtained in the form of pure cellulose or lignocellulose biomass consisting of hemicellulose, lignin, and other extractives [21]. Cellulose fibers have high strength and durability toward water and chemicals and have been utilized for paper and textile manufacturing [22]. According to its crystallinity, particle size, morphology, and degree of polymerization, cellulose material can be classified into macro- and nano-scale materials, with the most common cellulose used being microcrystalline cellulose (MCC) and nanocrystalline cellulose (NCC) [23,24]. MCC produces high mechanical properties with an elastic modulus of ~25 GPa and a high degree of crystallinity in the range of 55–80% [25]. In comparison, NCC has higher crystallinity and mechanical properties with an elastic modulus of ~167.5 GPa [26]. These cellulose properties are comparable with commercial aramid paper and can potentially be developed as a high-performance yet environmentally friendly paper.

Studies regarding high-performance paper from cellulose material in very diverse applications have been extensively reported. Kim et al. studied electro-active paper based on cellulose material and resulted in a high Young’s modulus in the range of 4–9 GPa [27]. Huang et al. obtained a high mechanical performance of graphene oxide/cellulose paper with excellent gas barrier properties for a packaging application [28]. Cellulose also has been studied as an environmentally friendly supercapacitor for a high-performance energy storage device by Huang et al. [29]. Ratajczak et al. also reported the achievement of high-performance cellulose paper research for a medical biosensor application [30]. However, to the extent of our knowledge, the utilization of cellulose as a high-performance paper in structural applications, especially a honeycomb core material, has not yet to be reported.

Thus, in this research, we studied high-performance paper based on cellulose material from *Cladophora* algae. According to a previous study by Mihranyan, it was reported that *Cladophora* algae, a filamentous green alga, has a high degree of crystallinity, around 95%, which results in high mechanical properties and chemical resistance due to its inter- and intramolecular hydrogen bonds [31]. *Cladophora* algae are a type of algae that can be found in fresh and briny water [32]. Moreover, the uncontrolled growth of *Cladophora* algae, called “algae blooms”, will cause pollution and disrupt the natural balance of the ecosystem [33,34]. *Cladophora* algae cellulose has been reported to be potentially used in various applications such as hydrogel [35], as the separator material of a Li-ion battery [36], as conductive paper [37], and so on. However, the study of *Cladophora* algae as an NCC material and for structural applications is not widely reported.

In this work, cellulose was extracted from *Cladophora* algae and developed as an alternative material to substitute for aramid paper in a honeycomb core application. Cladophora algae cellulose-based paper was prepared as MCC and NCC materials in this research. Then, the paper was formed into a honeycomb core structure and impregnated with epoxy resin. Finally, the sandwich-structured composite was prepared by assembling unidirectional carbon-fiber face sheets between the honeycomb core produced. Furthermore, the mechanical properties of the constituent materials and the sandwich-structured composite were investigated. As a result, the honeycomb core based on *Cladophora* algae cellulose paper exhibited impressive results that showed relatively high out-of-plane mechanical properties yet was sustainable and environmentally friendly.

## 2. Materials and Methods

### 2.1. Materials

In this study, *Cladophora* algae were acquired from Karakal beach, Special Region of Yogyakarta, Indonesia. Sodium hydroxide (NaOH) and hydrogen peroxide (H_2_O_2_) were purchased from Bratachem Inc. (Bandung, Indonesia). Analytical grade sulfuric acid (H_2_SO_4_) was purchased from Sopyan Jaya Cemerlang Inc. (Bandung, Indonesia). Eposchon A&B (bisphenol A—epichlorohydrin epoxy resin and polyaminoamide hardener) and acetone were purchased from Justus Kimiaraya Inc. (Bandung, Indonesia).

### 2.2. MCC and NCC Extractions

#### 2.2.1. MCC Extraction

The extraction process of MCC was started by cleaning all the dirt off the *Cladophora* algae and it was washed using tap water. Then, the cleaned *Cladophora* algae were dried at room temperature. The dried *Cladophora* algae were treated with an alkali using 17.5% NaOH at 100 °C for 3 h. The residue was filtered, washed until neutral pH was obtained, and dried at room temperature. The resulting alkali-treated *Cladophora* algae were treated with acid using 1 M H_2_SO_4_ at 100 °C for 3 h. The residue was filtered, washed until a neutral pH was obtained, and dried at room temperature. The resulting acid-treated *Cladophora* algae were bleached using 5% H_2_O_2_ at 100 °C for 3 h. The residue was filtered and washed until a neutral pH was obtained. Then, it was dried at room temperature in a Petri dish.

#### 2.2.2. NCC Extraction

In this study, the preparation of NCC material was carried out using acid treatment on the previously extracted MCC. The dried MCC was subjected to acid treatment using 2, 3, and 5 M H_2_SO_4_ at 100 °C for various treatment times, i.e., 5, 10, and 15 h. The variation was conducted to determine the optimum processing parameters to produce suitable NCC materials for honeycomb paper utilization. Then, the residue was filtered using filter paper, washed until a neutral pH was obtained, and dried at room temperature. The treatment was followed by bleaching using 5% H_2_O_2_ at 100 °C. The residue was filtered using filter paper, washed until a neutral pH was obtained, and dried at room temperature. The details of the samples’ name are shown in Table 1.

### 2.3. Preparation of MCC and NCC Paper

The preparation of paper made of MCC and NCC material was carried out using the solution casting method in a 15 × 15 cm^2^ mold. The MCC paper was prepared in the grammage of 80, 100, 200, and 300 g/m^2^, while the NCC paper was prepared in the grammage of 80 and 100 g/m^2^. The extracted MCC or NCC was dispersed using 1% H_2_SO_4_ solution with vigorous stirring at 100 °C until the cellulose was well dispersed. The solution was filtered and the residue was washed until neutral pH. Then, the pulp was obtained. The distilled water was added to the pulp in a 1:100 (*w*/*w*) ratio and stirred until the suspension was well dispersed. Finally, the suspension was molded and dried at room temperature.

### 2.4. Preparation of Honeycomb Core

The honeycomb core with a hexagonal structure in a ±7 mm cell size was made from MCC and NCC paper. The samples were prepared manually using the paper folding (origami) method. After the paper was successfully formed into a honeycomb core shape, the samples were dipped into 55–60 wt% epoxy A/B (1:1, *w*/*w*) with the addition of acetone 50% for ±10 min and cured at room temperature.

### 2.5. Characterizations

#### 2.5.1. Scanning Electron Microscopy (SEM)

The morphology of native and alkali-treated *Cladophora* algae, MCC, and cross-section areas of MCC and NCC paper was observed using JSM 6510 SEM (JEOL Ltd., Tokyo, Japan) at Balai Besar Tekstil, Bandung, Indonesia. The samples were prepared and coated with platinum using the JEC-3000FC sputter coater (JEOL Ltd., Tokyo, Japan) and then used for imaging.

#### 2.5.2. Transmission Electron Microscopy (TEM)

The morphology of NCC was examined using HT770 TEM (Hitachi Ltd., Tokyo, Japan) at the Research Center for Nanoscience and Nanotechnology ITB, Bandung, Indonesia. NCC samples were prepared in suspension form. The suspension was diluted using distilled water with a concentration of 2 wt%. The suspension was sonicated and deposited onto a carbon-coated copper grid. The samples were dried and observed.

#### 2.5.3. X-ray Diffractometer (XRD)

The crystallinity of native *Cladophora* algae, MCC, and NCC was examined using a D8 Advance X-ray Diffractometer (Bruker, Billerica, MA, USA) at the Faculty of Mechanical and Aerospace Engineering Laboratory, ITB, Bandung, Indonesia. The samples were prepared and then subjected to XRD spectrum at the glancing angles (2θ) of 10–90°. The degree of crystallinity of the samples was calculated according to Hinrichen’s method using Equation (1).

(1) $$Crystallinity\left(\%\right)=\frac{area\;of\;all\;the\;crystalline\;peaks}{the\;total\;area\;of\;crystalline\;and\;amorphous\;areas}\times 100$$

#### 2.5.4. Tensile Test

The tensile test was performed to measure the tensile properties of MCC and NCC paper using Tensilon RTF-1310 (A&D Company, Tokyo, Japan) according to the TAPPI 949 standard at the Production Engineering Laboratory ITB, Bandung, Indonesia. The samples were cut to a length of 45 mm and a width of 6.25 mm. Two types of samples were prepared, which were non-impregnated paper and epoxy-impregnated paper. The impregnated paper was prepared by dipping the paper into epoxy resin at various acetone additions, i.e., 0%, 25%, and 50%, and then cured at room temperature. The test was conducted with the moving clamp speed of 6.25 mm/min and the measurement was repeated three times.

#### 2.5.5. Compression Test

A compression test was performed to measure the compression strength of the MCC and NCC-based honeycomb core using Tensilon RTF-1310 (A&D Company, Tokyo, Japan) according to the ASTM C635 standard at the Production Engineering Laboratory ITB, Bandung, Indonesia. Honeycomb core samples from the previous step were prepared in a sandwich construction using unidirectional (UD) carbon fiber/epoxy composite as the skin material. Then, the skin was attached to the honeycomb core with epoxy resin as the adhesive. The compression test was conducted with a speed test of 0.5 mm/min.

## 3. Results

### 3.1. (Microcrystalline Cellulose) MCC and (Nanocrystalline Cellulose) NCC Extractions

#### 3.1.1. Microcrystalline Cellulose (MCC)

**Figure 1:**
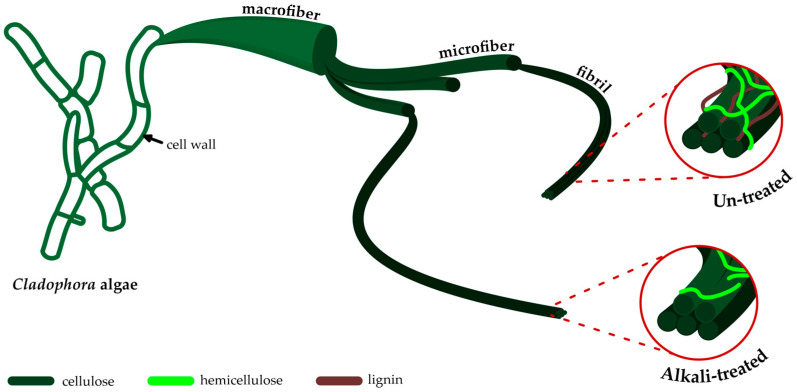
The illustration of the alkali treatment process to eliminate lignin, hemicellulose, and extractive contents from *Cladophora* algae.

MCC was prepared from *Cladophora* algae using an alkali treatment process to dissolve other substances, such as lignin, hemicellulose, and the remaining extractives. Sodium hydroxide (NaOH) from the alkali treatment broke down the linkage of those substances and increased the cellulose content in *Cladophora* algae. The yield produced from the alkalization process was around 66.09%, indicating the removal of lignin, hemicellulose, and extractive contents. The alkalization process of the lignocellulose is illustrated in Figure 1.

**Figure 2:**
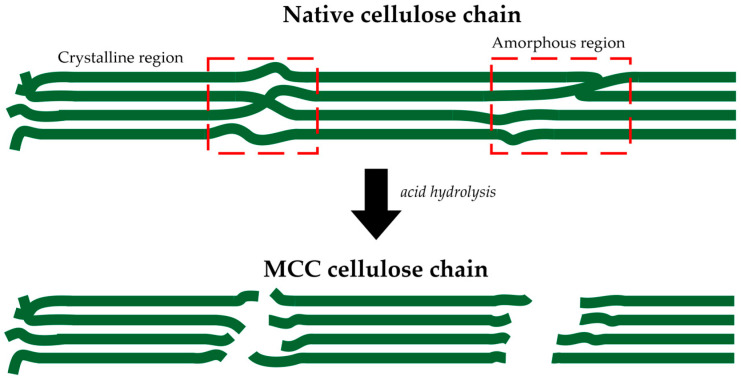
Acid hydrolysis treatment breaks down the cellulose linkage in the amorphous region.

The alkali-treated *Cladophora* algae were subjected to acid hydrolysis treatment using 1 M sulfuric acid to produce the MCC material. Acid hydrolysis treatment caused the breakdown of cellulose linkage in the amorphous region due to a sulfuric acid attack, resulting in a shorter cellulose chain than the native cellulose chain. The yield obtained from the acid hydrolysis process was 53.83%, proving the significant loss of the amorphous cellulose contents. The acid hydrolysis process to produce MCC material is illustrated in Figure 2.

**Figure 3:**
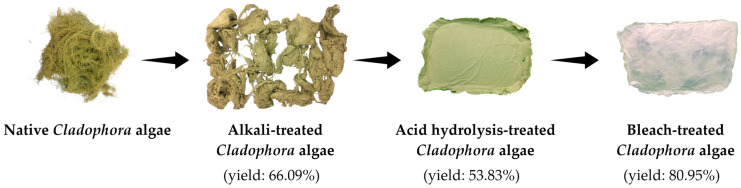
The appearance and yield obtained from the extraction process of MCC from *Cladophora* algae.

The next step was bleaching treatment to eliminate chlorophyll substances that give the green color pigment to the *Cladophora* algae. Bleaching treatment was conducted using hydrogen peroxide (H_2_O_2_). During alkalization treatment, the hydroxyl radical broke down the linkage of chromophores or chlorophyll substances along with the remaining lignin and hemicellulose [38]. The bleaching treatment produced a yield of around 80.95% due to the loss of chlorophyll substances. The resulting MCC was in paper form due to its strong hydrogen bonding that caused the cellulose fibril to be interlocked. The results of each treatment from the extraction process of MCC are shown in Figure 3.

#### 3.1.2. Nanocrystalline Cellulose (NCC)

**Figure 4:**
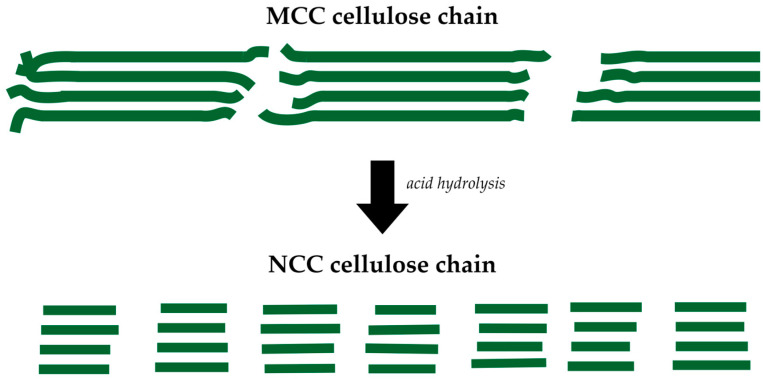
Acid hydrolysis treatment eliminates the amorphous chain to produce NCC material.

Acid hydrolysis treatment was conducted to produce the NCC material, using sulfuric acid with a higher concentration at various acid concentrations and hydrolysis times than the parameters used in the previously extracted MCC. Further acid hydrolysis treatment caused the MCC material to become darker due to cellulose chain depolymerization in the amorphous region. In contrast, the cellulose chain in the crystalline region was more susceptible to the sulfuric acid attack. Hydronium (H^+^) ions from the sulfuric acid react with oxygen from the glycosidic bond between glucose units and cause the chain scission of the glucose units within the amorphous cellulose region. In addition, the amorphous region in the cellulose fibril has numerous free volumes compared to the crystalline region, resulting in a higher number of hydronium ions that could penetrate into the amorphous region and break the glycosidic bond of the cellulose chain. Therefore, the cellulose chain becomes shorter due to the dissolved amorphous region during acid hydrolysis treatment. The acid hydrolysis process to produce the NCC material is illustrated in Figure 4.

### 3.2. The Morphology of MCC and NCC

#### 3.2.1. MCC

**Figure 5:**
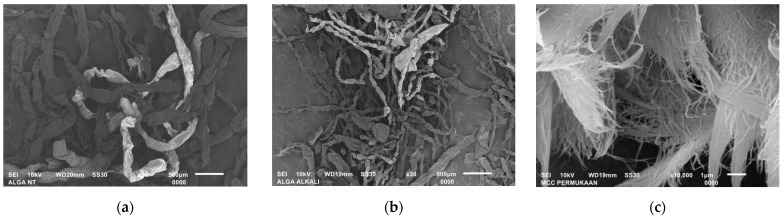
SEM images of the (**a**) native *Cladophora* algae, (**b**) alkali-treated *Cladophora* algae, and (**c**) the extracted MCC of *Cladophora* algae.

The extracted MCC morphology was examined using Scanning Electron Microscopy (SEM) characterization and the result is shown in Figure 5.

According to the SEM figures, it was confirmed that MCC was successfully extracted from the native *Cladophora* algae. Originally, the native *Cladophora* algae grew in a filament form with a width of ±196.46 µm. Following the alkali treatment process, the width reduced to ±73.93 µm, which indicated the dissolving of lignin, hemicellulose, and other substances. Subsequently, as a result of acid hydrolysis treatment, the MCC material demonstrated a greater number of fibril entanglements, otherwise known as a web-like structure, which were ±44.75 nm in width. In addition, it exhibited a long and dense fibril arrangement due to the numerous hydrogen bonds from the cellulose *Cladophora* algae source. The result indicated that the extracted MCC morphology was similar to the previous study reported by Mihranyan et al. [33]. According to this type of morphology, cellulose *Cladophora* algae was expected to have high mechanical properties and the potential to be used as a high-performance material.

#### 3.2.2. NCC

**Figure 6:**
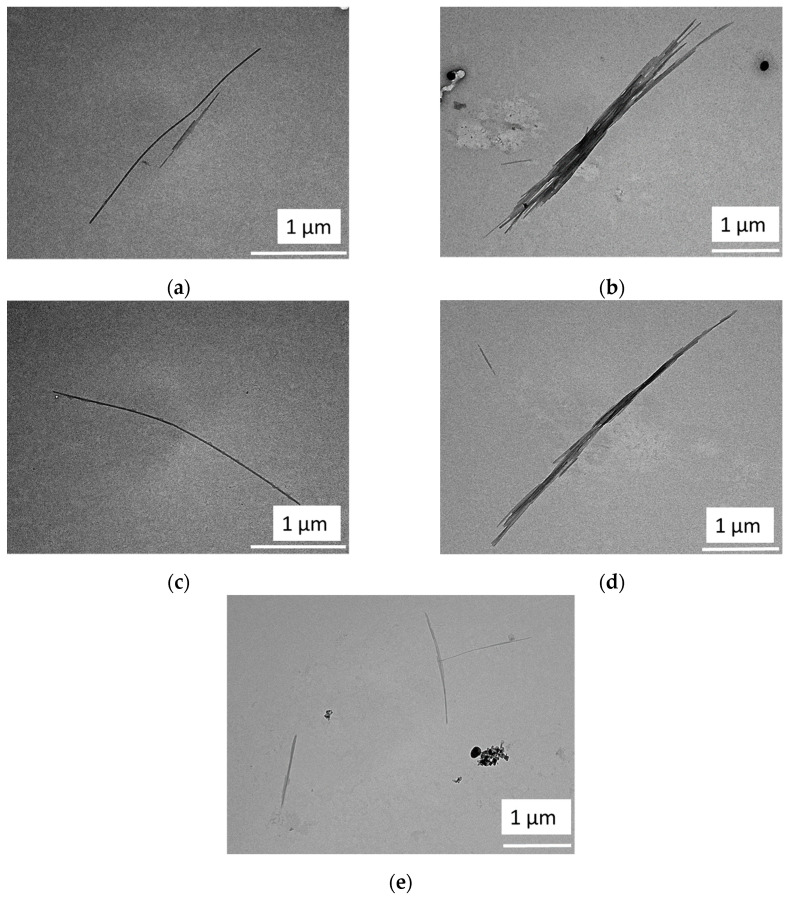
TEM images of the extracted NCC samples: (**a**) NCC2M15h, (**b**) NCC3M15h, (**c**) NCC5M5h, (**d**) NCC5M10h, and (**e**) NCC5M15h.

TEM images of the extracted NCC particle are shown in Figure 6. According to the TEM results, the NCC material exhibited needle-like particles which were 238 nm–8.5 µm in length and 14–25.5 nm in width. Due to the size range obtained, the extracted cellulose particles in this study can be classified as an NCC material (2–20 nm in width and 100 nm to several µm in length) [39]. During hydrolysis treatment, the higher acid concentration in the MCC material resulted in a shorter cellulose chain due to the amorphous region’s dissolution. The resulting morphology was similar to the reference reported by Sucaldito and Camacho [26]. The remaining cellulose chains were mostly in the crystalline region and were expected to have higher mechanical properties due to their numerous hydrogen bonds, creating a smaller pore size than the MCC material.

### 3.3. The Crystallinity of MCC and NCC

**Figure 7:**
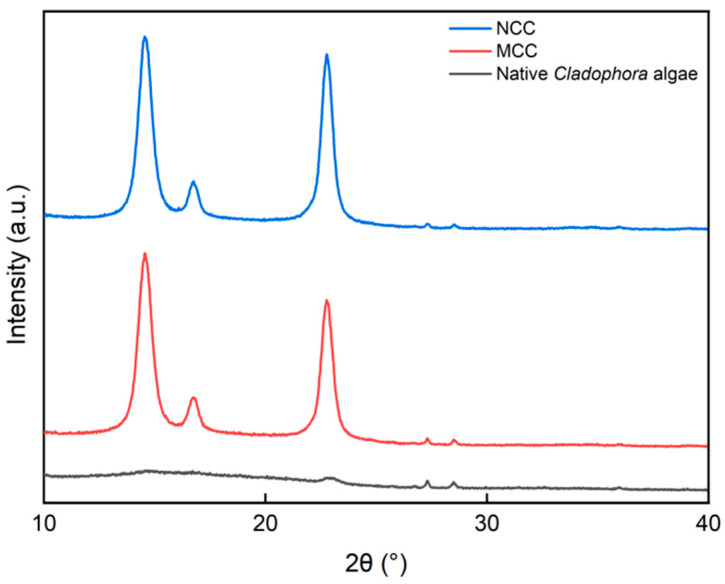
XRD pattern of native *Cladophora* algae, MCC, and NCC materials.

The crystallinity of the extracted MCC and NCC was determined using an X-ray diffractometer. The XRD pattern showed that the chemical treatment of the native *Cladophora* algae increased the degree of crystallinity of the extracted cellulose, as shown in Figure 7. The native *Cladophora* algae initially had a crystallinity of ±15.76%, which indicated that the native *Cladophora* algae is an amorphous material. In comparison, the MCC and NCC have a crystallinity of 76.92% and 94.84%, respectively. The results indicated that the amorphous region in the NCC nearly experienced a complete dissolution due to the hydrolysis treatment at a high acid concentration compared to the MCC.

### 3.4. MCC and NCC Paper

#### 3.4.1. MCC Paper

**Figure 8:**
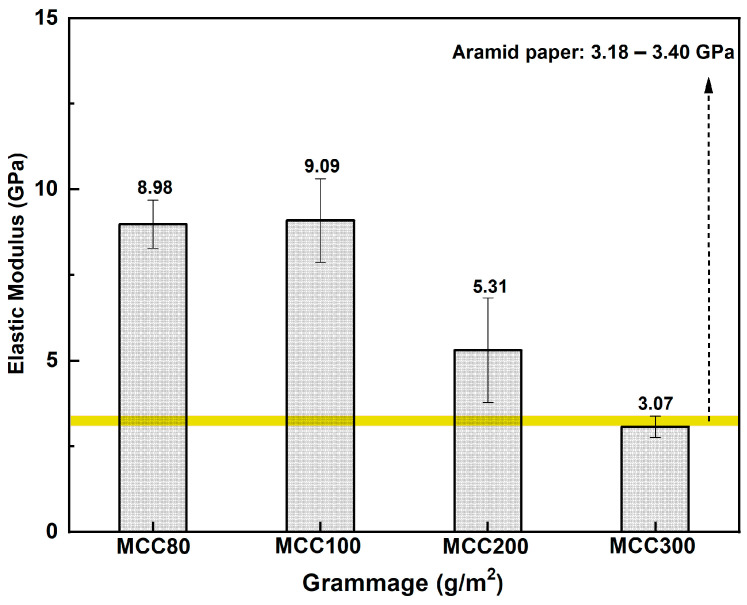
Elastic modulus of MCC paper at various grammages.

MCC paper was prepared at various grammages, i.e., 80, 100, 200, and 300 g/m^2^. The grammage affects the density and the distribution of cellulose fibrils to form the paper. The tensile strength of the MCC paper as compared to the aramid paper [40,41] is shown in Figure 8.

According to the tensile test result, the MCC100 paper showed an excellent elastic modulus result of 9.09 GPa, which exceeded the elastic modulus range of the commercial paper. It was followed by the MCC80 paper with an elastic modulus of 8.98 GPa. On the other hand, the MCC200 and MCC300 paper exhibited downward patterns with an elastic modulus of 5.31 and 3.07 GPa, respectively. It was likely due to the non-uniformity of fibril distribution within the paper because of the paper-making method. As a result, a certain part of the paper experienced stress concentration and caused paper failure before reaching its ultimate strength when subjected to a load. Thus, the paper-making method used needed to be more suitable for a higher grammage paper. Overall, it was observed that MCC paper exhibited relatively high mechanical performance owing to its web-like structure. The fibril entanglement produced numerous hydrogen bonds and resulted in a high elastic modulus of the MCC paper. Hence, it requires a large amount of energy to break the hydrogen bonding.

#### 3.4.2. NCC Paper

**Figure 9:**
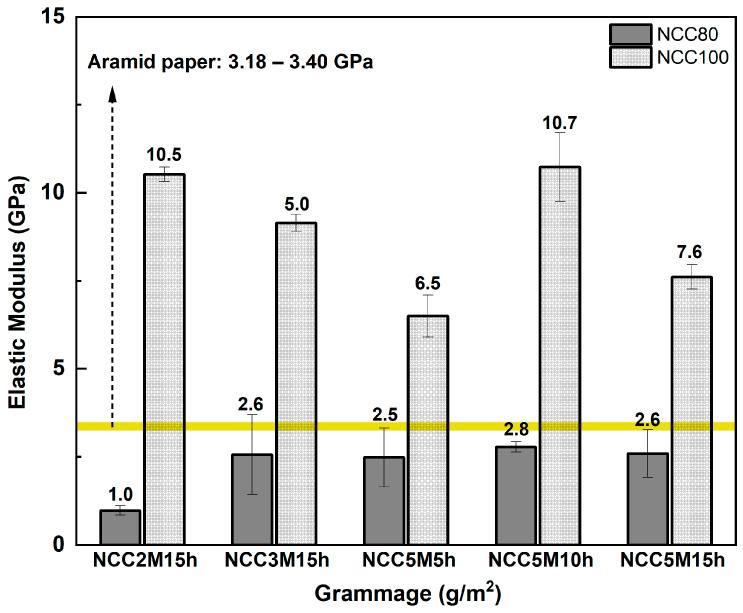
Elastic Modulus of NCC paper at various grammages.

NCC paper was fabricated using five types of NCC samples (NCC2M15h, NCC3M15h, NCC5M5h, NCC5M10h, and NCC5M15h). Each sample was prepared in 80 and 100 g/m^2^ grammages in accordance with the tensile test result of the MCC paper, whereas the optimum grammage used to produce high elastic modulus paper was MCC80 and MCC100. The NCC paper showed a smooth surface compared to the MCC paper due to its smaller cellulose particle dimension, creating denser fibril distributions. The elastic modulus of NCC paper samples compared to the aramid paper [40,41] is shown in Figure 9. The result showed that NCC5M10h with a grammage of 100 g/m^2^ exhibited the highest elastic modulus by 10.7 GPa. On the other hand, the elastic modulus of the NCC80 samples was significantly lower than that of the NCC100 samples. This is presumably due to the low amount of NCC particles present to retain the subjected load during tensile testing. In addition, NCC100 samples surpassed the elastic modulus range of the aramid paper.

### 3.5. Epoxy-Impregnated MCC and NCC Paper

#### 3.5.1. Epoxy-Impregnated MCC Paper

Prior to the preparation of the sandwich-structured composite, the paper used as the honeycomb core material was impregnated with epoxy resin. It was expected that the impregnation would enhance the stiffness of the honeycomb core material. MCC80 and MCC100 were selected to be impregnated with epoxy resin due to their high elastic modulus based on the previous tensile test result. MCC80 and MCC100 were impregnated with epoxy resin at various acetone addition concentrations, i.e., 0, 25, and 50%. The purpose of acetone addition into epoxy resin was to decrease its viscosity, resulting in better impregnation of the MCC paper. In comparison, the epoxy-impregnated MCC paper without acetone addition resulted in an opaque paper because of the high viscosity of epoxy resin to fill the paper pores.

**Figure 10:**
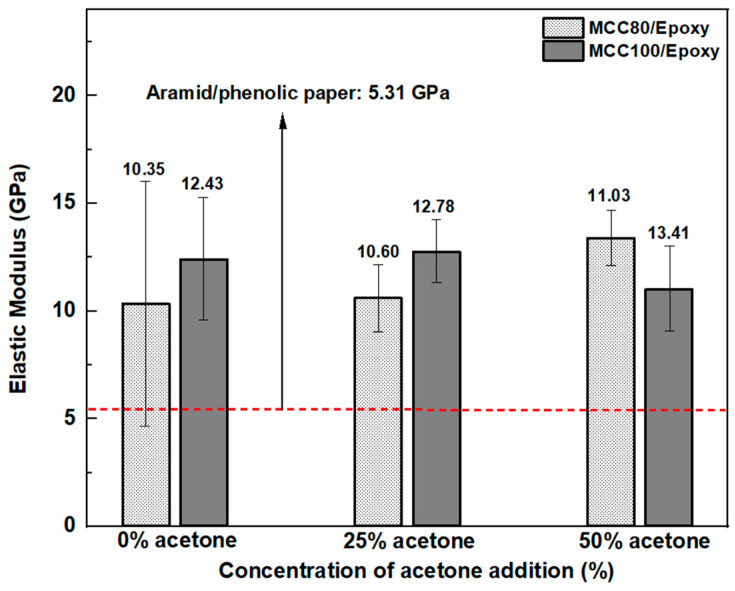
Elastic modulus of epoxy-impregnated MCC paper at various grammage.

The elastic modulus of the epoxy-impregnated MCC paper compared to the aramid/phenolic paper [40] is shown in Figure 10. Epoxy-impregnated MCC paper showed excellent results with a higher elastic modulus than the non-impregnated MCC paper and the phenolic-impregnated aramid paper. Epoxy resin reduced the paper’s porosity and stress was well distributed when the load was subjected. The graphic illustrates that MCC80, with 50% acetone addition to the epoxy resin, has the highest elastic modulus of 11.03 GPa. It was expected that the adequate viscosity of epoxy resin would fill the paper pores. In addition, the increased acetone concentration in epoxy resin reduced the epoxy viscosity and improved the cellulose paper’s impregnation. Thus, it increased the elastic modulus of the MCC paper.

#### 3.5.2. Epoxy-Impregnated NCC Paper

**Figure 11:**
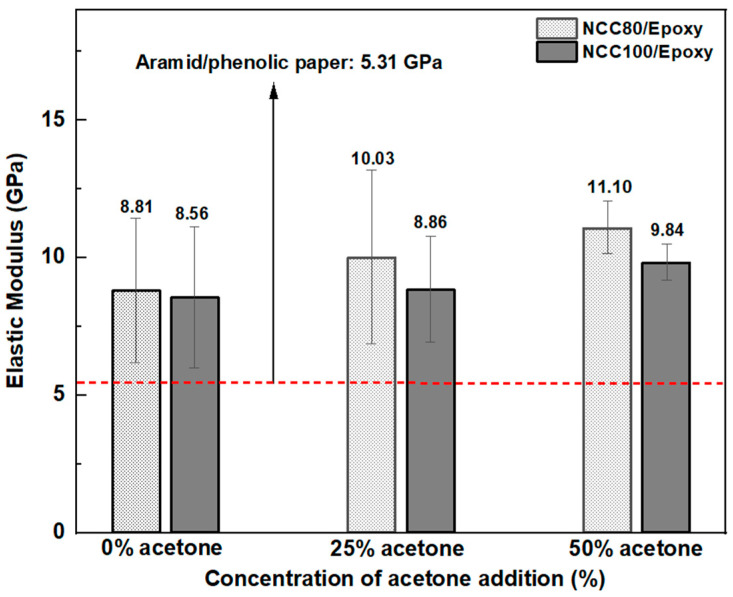
Elastic modulus of epoxy-impregnated NCC paper at various grammage.

According to the previous tensile test result, the optimum elastic modulus was achieved by the NCC5M10h sample. Therefore, that sample was chosen for the impregnation process. NCC paper with the grammage of 80 and 100 g/m^2^ was also impregnated with epoxy resin at various acetone addition concentrations, i.e., 0, 25, and 50%. Due to its denser fibril distribution than MCC paper, epoxy resin was more challenging to impregnate into the NCC paper. The tensile test results in comparison with the aramid/phenolic paper [40] are shown in Figure 11. The result demonstrated that the elastic modulus of the epoxy-impregnated NCC paper was higher than the non-impregnated NCC paper and the phenolic-impregnated aramid paper. In addition, the acetone addition improved the mechanical properties of NCC paper. Epoxy impregnation filled the pores of the NCC paper and improved its mechanical properties. Furthermore, the graphic illustrated that the epoxy-impregnated NCC80 paper with 50% acetone addition exhibited the highest elastic modulus by 11.10 GPa.

**Figure 12:**
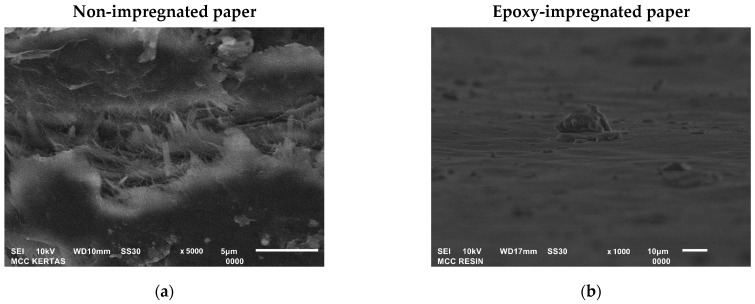

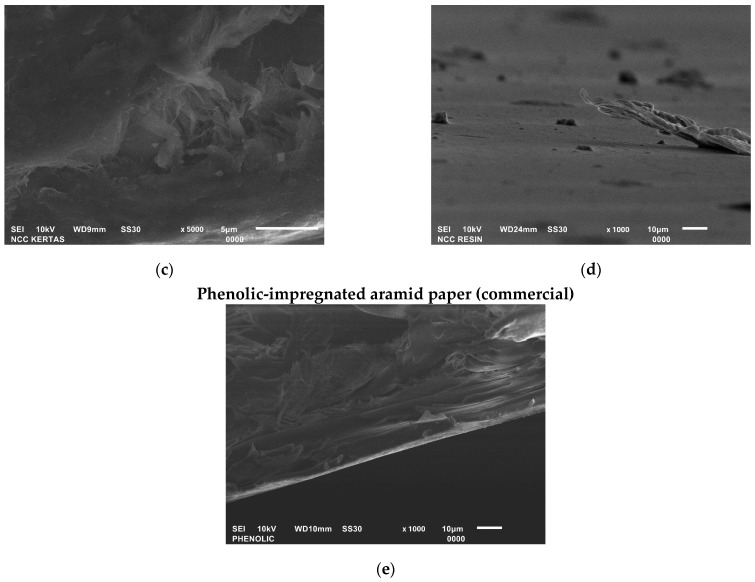
SEM images of the cross-section of (**a**) MCC paper, (**b**) epoxy-impregnated MCC paper (**c**) NCC paper, (**d**) epoxy-impregnated NCC paper, and (**e**) phenolic-impregnated aramid paper.

Overall, the impregnation of epoxy increased the elastic modulus of MCC and NCC paper. The epoxy resin filled in the voids within the MCC and NCC paper, which is confirmed by the SEM images in Figure 12. The cross-section area of epoxy-impregnated paper exhibited smoother surfaces compared with the non-impregnated paper. On the other hand, the phenolic-impregnated aramid paper showed a rougher morphology (see Figure 12e) at the cross-section area than the cellulose-based paper made in this study. As the result of the tensile testing, cellulose paper with a grammage of 80 g/m^2^ and epoxy resin impregnation displayed an outstanding elastic modulus compared to the other samples and the commercial one. Epoxy resin filled the vacant spots within the cellulose paper, resulting in preferable stress distribution when subjected to a load. In addition, the epoxy-impregnated cellulose paper showed a higher elastic modulus than the phenolic-impregnated aramid paper. The comparison of each constituent material for the honeycomb core application can be seen in Table 2.

### 3.6. The Compression Strength of MCC- and NCC-Paper-Based Honeycomb Core

**Figure 13:**
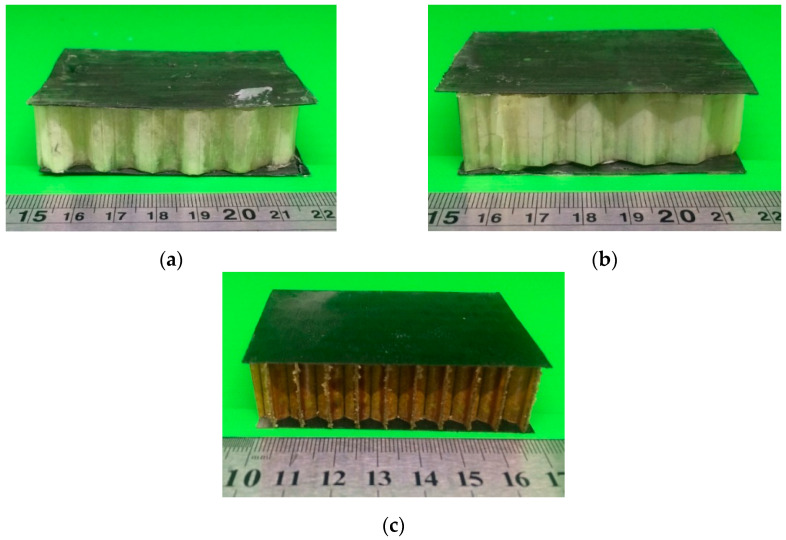
Out-of-plane compression test samples of sandwich-structured composite using UD carbon-fiber face sheets in various types of honeycomb core: (**a**) MCC paper, (**b**) NCC paper, and (**c**) aramid paper/commercial honeycomb core.

The honeycomb core was prepared using MCC and NCC paper with the optimum elastic modulus obtained in accordance with the previous tensile test result. Due to its excellent mechanical properties, MCC and NCC paper with 80 g/m^2^ grammage was chosen for the honeycomb core’s constituent material with 50% acetone-added epoxy resin. MCC and NCC paper was prepared into a hexagonal structure with a ±7 mm cell size using a manual method. Then, the out-of-plane compression test was conducted and compared with the aramid-paper-based honeycomb core. The samples of the out-of-plane compression test are shown in Figure 13.

**Figure 14:**
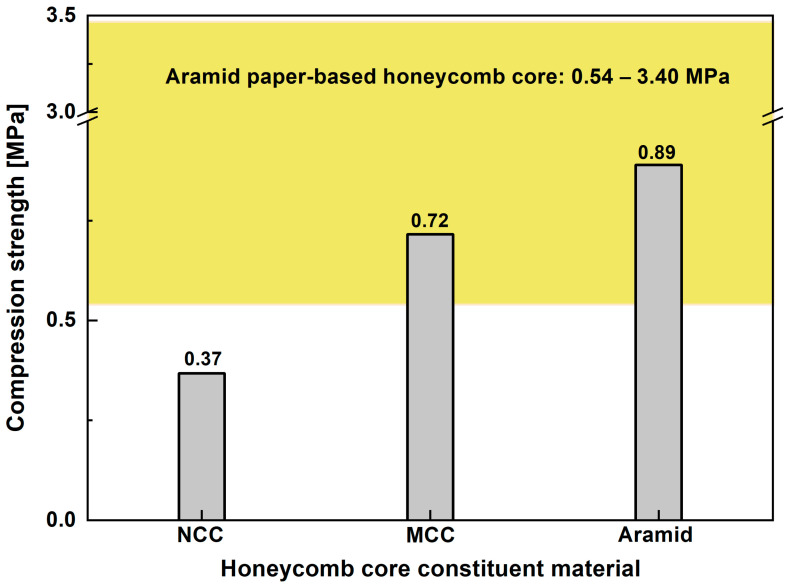
The out-of-plane compression strength of the honeycomb core of MCC, NCC, and aramid paper.

The result of the out-of-plane compression test is shown in Figure 14. The honeycomb core’s compression strength based on the MCC paper was higher than the NCC paper, which was 0.72 MPa. However, the compression strength of the aramid-paper-based honeycomb core sample was still higher due to the fact that the MCC-paper-based one was manually made and produced a rudimentary product. Nevertheless, the MCC-paper-based honeycomb core’s compression strength is in the commercial honeycomb core range with a comparable cell size (6.4 mm) [42]. When the honeycomb samples were subjected to the compression test load, the failure was indicated by buckling in the out-of-plane direction. It showed that the honeycomb core was unable to maintain its shape due to the given load. In this case, the elastic modulus property plays an important role in resisting load for the honeycomb core application. Hence, the compression properties are considered as one of the most important factors for the honeycomb core in a sandwich-structured composite.

## 4. Conclusions

MCC and NCC material was successfully extracted from *Cladophora* algae and utilized as high-performance paper for honeycomb core materials for sandwich-structured composite applications. As a result of the extraction process, MCC material resulted in the morphology of a web-like structure with numerous entanglements within the cellulose fibrils. On the other hand, the extraction of NCC produced a rod-like fibril morphology with a size of 238 nm–8.5 µm in length and 14–25.5 nm in width. MCC and NCC paper were prepared at various grammages and it was shown that epoxy-impregnated MCC paper exhibited a higher elastic modulus than NCC paper due to its morphology. The epoxy-impregnated MCC and NCC paper with the highest elastic modulus of 13.41 GPa and 11.40 GPa were prepared for the honeycomb core applications. The results showed that the epoxy-impregnated MCC paper experienced a higher compression strength than the epoxy-impregnated NCC paper, which was 0.72 MPa. In summary, several benefits can be drawn from this study: (a) cellulose material can be used as an alternative material for honeycomb core applications with comparable properties to commercial ones, and (b) it produces an environmentally friendly honeycomb core material using low-cost and sustainable resources.

## Figures and Tables

**Table 1:** The sample names of NCC materials at various acid concentrations and hydrolysis times.

Sample’s Name	Acid Concentration [M]	Hydrolysis Time [h]
NCC2M15h	2	15
NCC3M15h	3	15
NCC5M5h	5	5
NCC5M10h		10
NCC5M15h		15

**Table 2:** Elastic modulus of the constituent material for cellulose-based honeycomb core.

Non-Impregnated Paper		
Paper	E [GPa]	Source
MCC80	8.98	Experiment
NCC80	2.80	Experiment
Aramid	3.18–3.40	[40,41]
Resin-impregnated paper		
MCC80/Epoxy	13.41	Experiment
NCC80/Epoxy	11.10	Experiment
Aramid/Phenolic	5.31	[40]

## Data Availability

Data presented in this study are available on request from the corresponding author.
